# How Do *Drosophila* Stay Awake during the Daytime? Dopaminergic Neurons Are Inhibited by the Pigment Dispersing Factor Signaling Pathway to Promote Wakefulness

**DOI:** 10.1523/ENEURO.0348-18.2018

**Published:** 2018-09-11

**Authors:** Rosalind S.E. Carney

## Abstract

**Highlighted Research Paper:** Wakefulness Is Promoted during Day Time by PDFR Signalling to Dopaminergic Neurons in *Drosophila melanogaster*, by, Sheetal Potdar and Vasu Sheeba

Sleep is regulated by the circadian clock so that organisms can best function with respect to their local environment. Significant insight into circadian clock circuitry and associated neurotransmitter functions have enabled the development of treatments for individuals who have difficulties with sleep onset, sleep maintenance, or arousal from sleep. However, less is currently known about the neural mechanisms that maintain wakefulness during the daytime. Narcolepsy, a chronic sleep disorder characterized by excessive daytime sleepiness, affects around one in every 2000 people in the United States (U.S. National Library of Medicine, 2018). Narcoleptics can have difficulty adhering to conventional working hours and maintaining societal interactions. Therefore, it is important to ask what are the neural mechanisms that promote the maintenance of wakefulness during the daytime.

*Drosophila melanogaster* is a useful model for this question because they have regular sleep bouts during the daytime and exhibit sleep behavior similarities to mammals (Hendricks et al., 2000; Shaw et al., 2000). Sleep behavior can be monitored by placing *Drosophila* in glass tubes in which activity is recorded by infrared beam. Pigment dispersing factor (PDF) is a circadian neuropeptide that is known to promote wakefulness in *Drosophila*. In their *eNeuro* publication, Potdar and Vasu (2018) performed a wide array of experiments in *Drosophila* to identify the downstream targets and signaling interactions of the PDF receptor (PDFR).

A prior study in *Drosophila* showed that *pdfr* ^5304^ and *pdfr* ^3369^ loss-of-function mutations increased sleep during the daytime and nighttime (Chung et al., 2009). When Potdar and Vasu backcrossed the *pdfr* ^5304^ and *pdfr* ^3369^ mutants over seven or eight generations to the *Iso31* (*w* ^1118^) background, they found that sleep was increased during the daytime only. This increase in daytime sleep was replicated during the subjective daytime when the *pdfr* mutants were transferred to constant darkness. Furthermore, compared to controls, both *pdfr* mutants also fell asleep sooner and slept longer within a typical daytime sleep bout, although the number of total sleep bouts was similar in all groups. Taken together, these observations indicate that in the absence of functional PDFR, daytime sleep is initiated sooner, is more consolidated, and is of longer duration.

Potdar and Vasu next altered *pdfr* expression in 26 *GAL4* lines representing distinct neuronal subsets that included circadian pacemakers, higher-order processing centers, or neuronal groups based on neurotransmitter expression. However, sleep was altered consistently in only a few of these lines. From these data, Potdar and Vasu hypothesized that circadian clock neurons may not be the downstream targets of the PDFR signaling pathway that promotes wakefulness during the daytime. However, further analyses showed that decreasing PDFR signaling to dopaminergic neurons increased day-time sleep, whereas increasing PDFR signaling to dopaminergic neurons suppressed day-time sleep and made it fragmented, in addition to delaying sleep onset. These observations suggest that dopaminergic neurons are the downstream targets of the PDFR signaling pathway that initiates and maintains wakefulness during the daytime.

A prior study had shown that dopamine acts on large ventral lateral neurons (l-LNv) to promote wakefulness (Shang et al., 2011). To determine whether PDF+ neurons synapse with dopaminergic axons, Potdar and Vasu used a GFP reconstitution across synaptic partners (GRASP) experiment. GRASP is a system in which two complementary GFP fragments, expressed on different cells, reconstitute as a GFP florescence reporter only at the site of a synapse (Feinberg et al., 2008). When the two GFP fragments were expressed in PDF+ and dopaminergic neurons, staining with an anti-GFP antibody revealed colocalization in the ascending portion of small lateral ventral neuron (s-LNv) dorsal projections, confirming synaptic connections (Fig. 1). s-LNv have been proposed to have an auxiliary role in the wake-promoting effects of l-LNv (Parisky et al., 2008; Potdar and Sheeba, 2012). Therefore, Potdar and Vasu examined the effects on sleep when a neurodegenerative protein was used to selectively render s-LNv dysfunctional (Sheeba et al., 2010), while simultaneously changing the electrical properties of l-LNv (Nitabach et al., 2006). These results validated that l-LNv modulate wakefulness and further show that functional s-LNv are required to mediate these effects.

**Figure 1.**
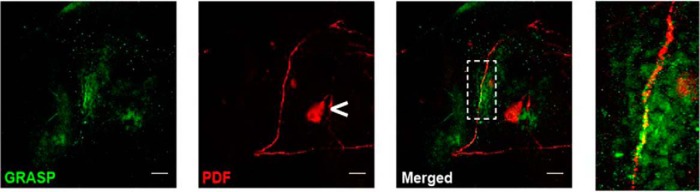
Anatomic connections between PDF+ neurons and dopaminergic neurons. GRASP signal (green) colocalized with the ascending portion of s-LNv dorsal projections labeled with an antibody against PDF (red).

In addition, by measuring calcium levels as indicators of neuronal activity, Potdar and Vasu showed that dopaminergic neurons, the likely targets of PDFR signaling, are less active during daytime. As PDF levels are high during the daytime, it is likely that the dopaminergic neurons that mediate wakefulness are, in fact, sleep-promoting and inhibited by PDF. These findings have identified a circadian clock pathway that promotes wakefulness during the daytime in *Drosophila*.
